# Supplementary material for: Smooth Interpolating Curves with Local Control and Monotone Alternating Curvature
Source: Comput Graph Forum. 2022 Oct 6;41(5):25–38. doi: 10.1111/cgf.14600 (PMC9827861; doi:10.1111/cgf.14600)
Supplement: Supplementary file 1 — Supplement Material [file CGF-41-25-s001.zip › Local-Smooth-Interpolating-MonoCurvature/extern/clothoids/docs/api-matlab/unabridged_orphan.html]

Full API — Clothoids v2.0.9

### Navigation

- index
- toc
- Clothoids »
- Full API

# Full API¶

## Files¶

- File Biarc.m
  - Definition (`Biarc.m`)
    - Program Listing for File Biarc.m
  - Classes

- File BiarcList.m
  - Definition (`BiarcList.m`)
    - Program Listing for File BiarcList.m
  - Classes

- File CircleArc.m
  - Definition (`CircleArc.m`)
    - Program Listing for File CircleArc.m
  - Classes

- File ClothoidCurve.m
  - Definition (`ClothoidCurve.m`)
    - Program Listing for File ClothoidCurve.m
  - Classes

- File ClothoidList.m
  - Definition (`ClothoidList.m`)
    - Program Listing for File ClothoidList.m
  - Classes

- File ClothoidSplineG2.m
  - Definition (`ClothoidSplineG2.m`)
    - Program Listing for File ClothoidSplineG2.m
  - Classes

- File CompileOctave.m
  - Definition (`CompileOctave.m`)
    - Program Listing for File CompileOctave.m

- File CurveBase.m
  - Definition (`CurveBase.m`)
    - Program Listing for File CurveBase.m
  - Classes

- File LineSegment.m
  - Definition (`LineSegment.m`)
    - Program Listing for File LineSegment.m
  - Classes

- File PolyLine.m
  - Definition (`PolyLine.m`)
    - Program Listing for File PolyLine.m
  - Classes

- File Triangle2D.m
  - Definition (`Triangle2D.m`)
    - Program Listing for File Triangle2D.m
  - Classes

- File TriTriOverlap.m
  - Definition (`TriTriOverlap.m`)
    - Program Listing for File TriTriOverlap.m
  - Functions

### Quick search

### Table of Contents

- Matlab Interface Manual
- C++ API
- MATLAB API

«
hide menu

menu
sidebar
»

### Navigation

- index
- toc
- Clothoids »
- Full API

© Copyright 2021, Enrico Bertolazzi and Marco Frego.
Created using Sphinx 4.2.0.
